# Hypertensive Patients Show Delayed Wound Healing following Total Hip Arthroplasty

**DOI:** 10.1371/journal.pone.0023224

**Published:** 2011-08-11

**Authors:** Awad A. Ahmed, Pekka A. Mooar, Matthew Kleiner, Joseph S. Torg, Curtis T. Miyamoto

**Affiliations:** 1 Temple University School of Medicine, Philadelphia, Pennsylvania, United States of America; 2 Department of Orthopaedics and Sports Medicine, Temple University Hospital, Philadelphia, Pennsylvania, United States of America; 3 Department of Radiation Oncology, Temple University Hospital, Philadelphia, Pennsylvania, United States of America; University of Michigan, United States of America

## Abstract

**Background:**

Prolonged wound-discharge following total hip arthroplasty (THA) is associated with an increased risk of infection. However, the potential role of hypertension in prolonging the duration of wound healing in this population has not yet been investigated. The aim of the present study was to compare healing in this population that has not yet been investigated. The aim of the present study was to compare hypertensive and normotensive THA patients in terms of the length of time required to achieve a dry wound and the length of stay in the hospital.

**Methods:**

One hundred and twenty primary THA patients were evaluated. Pre-operative clinical history and physical examination revealed that 29 were hypertensive and 91 were normotensive. The two groups were statistically matched using optimal propensity score matching. The outcomes of interest were the number of days until a dry wound was observed and the duration of hospital stay.

**Results:**

The average systolic blood pressures were 150.1 mmHg and 120.3 mmHg for the hypertensive and normotensive groups, respectively. The mean number of days until the wound was dry was 3.79 for the hypertensive group and 2.03 for the normotensive group. Hypertensive patients required more days for their wounds to dry than normotensive patients (odds ratio  = 1.65, p<0.05). No significant difference in the duration of hospital stay was found between the two groups.

**Conclusions:**

Hypertensive patients had a higher risk of prolonged wound discharge after THA than their normotensive counterparts. Patients with prolonged wound drainage are at greater risk for infection. Clinicians should pay particular attention to infection-prevention strategies in hypertensive THA patients.

## Introduction

A total of 230,000 total hip replacement procedures were performed in the United States in 2007 [1]. This number is expected to increase in the future due to outstanding long-term results and advances in prosthetic design, surgical technique, and instrumentation, as well as the existence of an aging population [2], [3]. Hanssen and Rand investigated a cohort of 23,519 patients who underwent total hip or knee arthroplasty between 1969 and 1996, and reported an infection rate of 1.3% [4]. The cost per case of post-operative infection has been estimated to be $44,935 [3], resulting in an annual cost to the US healthcare system of approximately $134,000,000.

Several studies have reported that persistent post-operative wound discharge was a significant predictor of infection following total joint arthroplasty [3], [5]–[7]. Estimates of post-operative infection are as high as 50% for patients with persistent wound discharge [6]. Few studies have investigated predictors of and risk factors for prolonged wound drainage. However, it has been shown that morbid obesity and the use of low molecular weight heparin as thromboembolic prophylaxis are significant risk factors [6].

**Figure 1:**
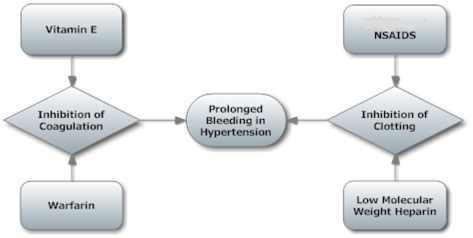
Interactions. Possible interactions in hypertensive patients undergoing THA that may contribute to delayed wound healing.

In 2005–2006, 32% of adults in the USA above the age of 20 years suffered from hypertension [8]. Hypertension is closely correlated with body mass index (BMI). To our knowledge, no previous study has investigated the effects of hypertension on prolonged wound discharge following THA. Examination of this relationship is indicated since cardioprotective medications or diets endemic to hypertensive populations may have potentiating or synergistic effects on coagulation or clotting cascades, particularly when used in conjunction with thromboembolic chemoprophylaxis. The rate of both nonfatal and fatal cardiovascular disease is directly proportional to systolic and diastolic blood pressure levels [9]. Aspirin and other non-steroidal anti-inflammatory drugs are commonly used prophylactic agents with effects on the clotting cascade. In addition, vitamin E has cardioprotective antioxidant properties [10], and hypertensive patients may thus choose to include foods rich in this vitamin in their diet. However, increased levels of vitamin E can be detrimental to these patients by prolonging bleeding time when combined with warfarin [11], and higher blood serum levels of this vitamin have been associated with higher systolic blood pressure levels [12]. These factors may also affect the bleeding time in hypertensive THA patients. When combined with chemoprophylaxis for thromboembolism, a therapy which also inhibits coagulation and clotting cascades, these agents may have effects on bleeding which may, in turn, lead to delayed wound healing. These interactions are summarized in Figure 1.

The aim of the present study was to examine the influence of hypertension on the length of time required until a wound is dry, and the duration of hospital stay. This was accomplished by examining two groups of patients with similar profiles in terms of factors with a potential impact on wound healing, but different blood pressure profiles.

## Methods

The present retrospective observational study was approved by the Institutional Review Board (IRB) at Temple University Hospital. Patient consent was not required since the study involved the evaluation of existing data, documents, and records and the anonymous recording of data by the investigator. Thus, individual subjects cannot be identified, either directly or through identifiers linked to the subject. Prospectively collected data from 121 consecutive primary THA procedures performed at our academic medical center from January 1, 2008 to December 31, 2009 were evaluated. Relevant data were extracted from patient charts. One patient was not included in this study because of insufficient data. Blood pressure values were obtained from data concerning clinical history and physical examination obtained within the 35 days prior to surgery. Discharge notes were used to obtain data on postoperative hospital course, and the nursing notes were accessed to obtain data on wound status. Hypertension was defined as a systolic blood pressure equal to or above 140 mmHg or a diastolic pressure equal to or above 90 mmHg, in accordance with the definition proposed by the Joint National Committee on Prevention, Detection, Evaluation, and Treatment of High Blood Pressure [9]. To minimize noise due to “white coat” hypertension [13], a past diagnosis of hypertension was also included in the definition used in the study, i.e., patients were only defined as hypertensive when they had presented with hypertension at the preoperative physical examination and had a previous diagnosis of hypertension.

Before optimal matching, the normotensive group included 91 patients and the hypertensive group included 29 patients. The two groups were adjusted using optimal matching and variables identified in previous studies as risk factors for wound healing complications (Table 1). The optimal matching method was used to minimize the effects of confounding factors. In particular, adjustment was made for BMI and diabetes in view of their close correlation with hypertension. Thromboembolic chemoprophylaxis was also adjusted for since this is an important factor in predicting prolonged wound secretion according to the previous literature and anecdotal evidence from the surgeons at our institution. After matching, each group included 29 patients.

**Table 1:** 

Variable			Significant Risk Factor
			in These Studies
Age			[31] *
Diabetes			[32] *
Gender			[31], [33] *
BMI			[31], [34] * [6] ‡
Thromboembolic Chemoprophylaxis			[6] ‡

*Denotes studies that examined infection as an outcome.

‡ Denotes studies that examined prolonged wound secretion as an outcome.

### Wound Closure

Following the total hip replacement procedure, the wound was closed by the insertion of deep heavy (0 or 1–0) vicryl sutures in the capsule and fascial layers, and interrupted 2–0 vicryl sutures in the subcutaneous layer. The wound was dressed with a compression style dressing using Abdominal (ABD) pads and tape, and the skin incision was closed with staples. The wound was covered until the second post-operative day, at which time-point daily inspection of the wound by a resident or attending surgeon was commenced. The dressing was first changed on the second post-operative day, and then daily on all subsequent days if active drainage was observed. Bandages were also changed daily from the second post-operative day. In cases where increased wound leakage was observed by the nursing staff, a resident or attending surgeon was alerted. Hip spica compression dressings were applied to all wounds showing active leakage, until dry. All wound dressings were changed by a resident or attending surgeon only. Evidence of serous or bloody drainage was recorded. In accordance with previous studies, active drainage was defined as drainage that substantially wetted or soaked a minimum gauze dressing surface area of 2×2-cm; and which emanated from the same specific site(s) along the wound [6], [14]. Wounds that were noted to be clean, dry, and intact by the nursing staff following the resident or attending surgeon's inspection were defined as being non-actively draining.

### Optimal Matching

The propensity score, defined as the conditional probability of being assigned to a particular condition given the observed background covariates, was initially defined by Rosenbaum and Rubin [15]. The rationale for the use of propensity scores can be understood by considering the ideal situation in which the variable and control groups are similar in terms of all background characteristics (as can be attained in a randomized experiment) [16]. Propensity scores are used to reduce selection bias by equating groups on the basis of these covariates. Thus, assuming no hidden bias, any difference in outcomes between these pairs can be attributed to the variable of interest rather than to any other difference between the variable and the control individuals [16].

The present study was a typical nonexperimental retrospective study. Since blood pressure levels are not randomly assigned to patients, more rigorous causal inference arguments are required to draw the conclusion that high blood pressure has an impact on the outcomes. The optimal matching algorithm [17], which determines the matched samples with the smallest average absolute distance across all the matched pairs, was implemented. Previous research has shown that while matching approaches generally involve the selection of the same sets of controls for the overall matched samples, optimal matching is superior in terms of minimizing the distance within each pair [18]. Furthermore, optimal matching is helpful when appropriate control matches for the units with the condition of interest are limited.

The propensity score for each patient was defined as the estimated logit function of the logistic regression of systolic blood pressure level on the following variables: age, BMI, history of diabetes, and gender. All THA patients at Temple University Hospital receive oral warfarin on the day of the procedure, and some patients are administered low molecular weight heparin (enoxaparin) subcutaneously on the first post-operative day at the discretion of the responsible surgeon. Anecdotal evidence from the surgeons at our institution and evidence reported in the literature [6] suggest that the use of enoxaparin was the strongest risk factor for delayed wound healing and should therefore be controlled for. Thus, to achieve optimal matching, separate matching was performed for this particular variable. The ratio for optimal matching was set at one. Following propensity score matching, each hypertensive patient receiving enoxaparin was matched with one normotensive patient receiving enoxaparin. This procedure was also performed for the hypertensive and normotensive patients who were not receiving enoxaparin. A total of 29 patients remained in the normal blood pressure group.

The optimal matching method using the *MatchIt* function in *R* by Imai was implemented [19]. The balance of matching was checked using the criteria proposed by Rubin [20]:

1. The standardized difference of means of the propensity score.
2. The ratio of the variances of the propensity score in the treated and control groups.
3. For each covariate, the ratio of the variance of the residuals orthogonal to the propensity score in the treated and control groups.

Prior to matching, large discrepancies in the variables of interest were apparent between the hypertensive and normotensive patients (Tables 2 and 3). After matching, all standardized differences of means were less than 0.25, variance ratios were between 0.5 and 2, and residual variance ratios for all covariates were between 0.5 and 2, which are the standards suggested by [20] for accurate regression adjustment after matching. Although high ratios for optimal matching were also attempted, no satisfactory matching balance could be achieved with ratios greater than one. Thus, only the advantages and results of a matching ratio equal to one are reported.

**Table 2:** 

Variable			Patients with Normal	Patients with Normal	Hypertensive Patients
			Blood Pressure	Blood Pressure	
			(Before Adjustment)	(After Adjustment)	
Age			58.3 (29–85)	62.1 (32–83)	61.3 (46–77)
BMI			30.0 (17.7–50.3)	31.7 (20.7–50.3)	32.0 (19.4–45.6)
Diabetes			23%	35%	31%
Gender (male)			50%	76%	72%
Mean Days to Dry Wound‡			1.60 (0–12)	2.03 (0–12)	3.79 (0–31)
Mean Days to Discharge‡ *			4.67 (2–30)	4.89 (3–14)	4.58 (2–23)
Readmission/Dehiscence (Patients)			0	0	5
Mean Blood Loss (Cubic Centimeters)			517 (225–1000)	550 (500–600)	540 (250–900)

‡ Durations <1 were assigned a value of zero.

*This only reflects the first admission and does not include days after readmission.

**Table 3:** 

	Standardized Difference of Mean		Ratio of Variance	Ratio of Residual Variance
	(Criteria 1)		(Criteria 2)	(Criteria 3)
	Before Matching	After Matching		
Age	1.154	0.076	1.41	1.43‡
BMI	0.125	0.0533	1.17	1.182‡
Diabetes	0.114	0.0728	1.06	1.116‡
Gender	0.758	0.078	1.09	0.938‡
Propensity Score	1.287	0.0311‡	1.06‡	0.004

‡ Values required for satisfying rigorous matching criteria.

To evaluate the number of days until the wound was dry and the duration of hospital stay, Poisson regressions of datasets were fitted before and after matching, and after adjustment for all of the variables used for matching.

## Results

**Figure 2:**
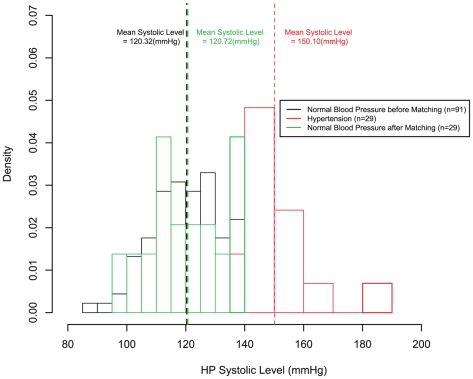
Distribution of the study population. Density as a function of systolic blood pressure levels. The average pressures were 120.6 mmHg, and 122.8 mmHg, for the normotensive group before matching, and after matching, respectively; and 152.2 mmHg for the hypertensive group.

**Figure 3:**
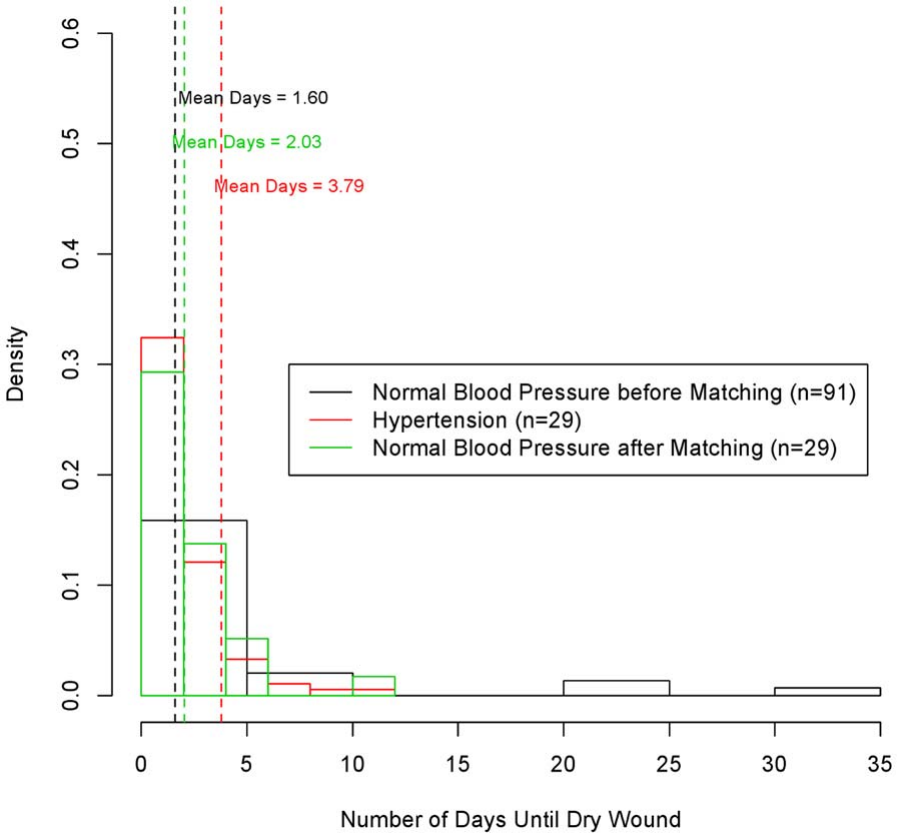
Blood pressure groups and days to dry wound. Density as a function of the number of days until a dry wound is observed. The mean number of days was 1.57, and 1.72 for the normal blood pressure group before matching, and after matching, respectively; and 4.32 for the hypertensive group.

Table 4( shows the odds ratios OR) for the Poisson model for high blood pressure levels. The wounds of hypertensive patients tended to require approximately 2 days longer to dry than those of normotensive patients. No significant difference was found between the groups in terms of the duration of hospital stay. Prior to adjustment, the control group had lower mean values for BMI, age, and incidence of diabetes (Table 2). Prior to adjustment, the difference in the number of days until the wound was dry was still significant, although the OR was slightly greater. Figure 2 shows the systolic blood pressure levels before and after matching. Figure 3 shows the profiles for the number of days until the wound was dry for all groups.

**Table 4:** 

Days to Dry Wound			Days Before Discharge	
All Data	After Matching		All Data	After Matching
2.52**	1.65**		0.92	0.94

**Denotes results with p-value <0.05.

## Discussion

The present study demonstrated that hypertension is associated with delayed wound healing following total hip replacement surgery. To our knowledge, this is the first report to address the issue of hypertension as a risk factor for prolonged wound discharge. The precise mechanisms underlying the delay in wound healing are unknown. However, the incidence of thromboembolic disease following THA is high [21]–[25], and pharmacological prophylaxis methods include antithrombotic and antiplatelet agents [26], [27]. Both warfarin and enoxaparin exert their effects by interfering with the coagulation or clotting cascade [26], [28]–[30]. Concurrent use of other clot-inhibiting agents may potentiate the delayed wound healing effect. Although the present study found no significant difference in the duration of hospital stay, patients with delayed wound healing require more care and are at higher risk of infection. It should be noted that all of the patients who had wounds that became infected were in the hypertensive cohort, although there were not enough to do a statistical analysis (n = 5).

These important considerations should be taken into account in the clinical management of hypertensive THA patients. The risk of thromboembolic disease following THA is high. However, the findings of the present study suggest that it may be advisable to use a compression device as an alternative to chemoprophylactic agents for hypertensive patients to avoid a greater complication risk. The effects of hypertension on other orthopaedic procedures should also be investigated. Although the clinical records used in the present study did not document blood vitamin levels, the present authors hypothesize that vitamin E may be implicated in delayed wound healing and thus warrant further investigation.

One limitation of the present study was the definition of blood pressure according to clinical history and the measurement taken during the pre-operative physical examination. Although this method was convenient, an average of past values might have been more reliable. However, defining which recorded measurements should be used to calculate the average would have been problematic since for some patients, records were limited. By contrast, the clinical history and pre-operative physical examination approach was a feasible and convenient choice for the entire cohort.

Hypertensive patients undergoing THA are at a greater risk for delayed wound healing and thus should be evaluated carefully. Further research should examine vitamin E intake, genetic factors, and environmental factors in patients that experience delayed wound healing.
